# Function of Transient Receptor Potential-Like Channel in Insect Egg Laying

**DOI:** 10.3389/fnmol.2022.823563

**Published:** 2022-06-30

**Authors:** Yan Zhang, Yi-Jie Zhang, Di Guo, Li-Xiang Wang, Chun-Dong Niu, Shun-Fan Wu, Yali V. Zhang, Cong-Fen Gao

**Affiliations:** ^1^College of Plant Protection, State and Local Joint Engineering Research Center of Green Pesticide Invention and Application, Nanjing Agricultural University, Nanjing, China; ^2^Monell Chemical Senses Center, Philadelphia, PA, United States

**Keywords:** *Nilaparvata lugens*, TRPL, RNAi, fecundity, egg-laying

## Abstract

The transient receptor potential-like channel (TRPL) is a member of the transient receptor potential (TRP) channel family involved in regulating many fundamental senses, such as vision, pain, taste, and touch, in both invertebrates and vertebrates. Yet, the function of TRPL in other important biological processes remains unclear. We discover that TRPL regulates egg laying in two insect species, the brown planthopper, *Nilaparvata lugens*, and the fruit fly, *Drosophila melanogaster*. In both insects, *trpl* is expressed in the female reproductive organ. Loss of *trpl* leads to significantly defects in egg laying. In addition, TRPL is functionally interchangeable between the brown planthoppers and flies in egg laying. Altogether, our work uncovers a novel role played by TRPL in regulating egg laying and indicates TRPL as a potential pesticide target in brown planthoppers.

## Introduction

Egg laying is a critical female reproductive behavior in insects and has a profound impact on the ecological success of insect species (Billeter and Wolfner, 2018). After mating, the female insects commit to laying eggs, during which external and internal cues are detected by the insects to trigger appropriate egg development and release. In general, females first localize a possible oviposition substrate from a distance, using olfaction and vision. Some contact-based sensations, such as touch and taste, are employed to stimulate egg laying when females land on the egg laying site. When the oviposition substrate is identified, the females complete the final stage of egg laying. The female reproductive tract is innervated by sensory and motor neurons relaying signals to the central nervous system to expel eggs out of the uterus (Turner et al., 2016; Cury et al., 2019).

Transient receptor potential (TRP) channels are a class of cation channels involved in sensory signaling (Ramsey et al., 2006; Venkatachalam and Montell, 2007). The TRP family consists of about 30 members in insects, which are further divided into 7 subfamilies: TRPC (canonical), TRPA (ankyrin), TRPM (melastatin), TRPV (vanilloid), TRPN (no mechanoreceptor potential), TRPP (polycystic), and TRPML (mucolipin). In many insects, the TRP channel family plays an indispensable role in the reproduction process. For instance, in flies TRPP2 is expressed in sperm flagella and is required for sperm to enter female fertilization vesicles (Kottgen et al., 2011). In *Bombyx*, TRPA1 is required for the detection of environmental temperature and affects the induction of diapause in progeny (Sato et al., 2014). Moreover, Painless, a TRPA subfamily member, plays an important role in controlling sexual receptivity (Sakai et al., 2014). Female flies employ the TRPV family channel Nanchung to evaluate the physical quality for egg-laying substrates (Zhang et al., 2020). The transient receptor potential-like (*trpl*) gene, a TRPC family member, was first identified in the fly photoreceptor cells, where it acts in concert with TRP to regulate phototransduction (Niemeyer et al., 1996; Xu et al., 1997). TRPL is also essential for calcium signaling in the epithelium (MacPherson et al., 2005). Moreover, TRPL is responsible for gustatory detection of bitter substances (Zhang et al., 2013). Given the emerging roles of the TRP family in many other physiological contexts, the requirement of TRPL in reproduction needs to be deciphered.

The brown planthopper, *Nilaparvata lugens*, is a severe rice pest in Asia (Liu and Han, 2006) that can cause extreme damage to rice by directly sucking the phloem sap, especially in the late growth period of rice. In 2005, it led to a rice harvest loss of 1.88 billion kg (Hu et al., 2011). The r-strategic brown planthopper has a relatively short life span and shows high fecundity, with each female laying 300–700 eggs (Ling et al., 2009, 2011). The biological control of this insect is of great urgency due to its high resistance to most insecticides. However, molecular mechanisms underlying the brown planthopper’s fertility remain poorly understood. The process of laying eggs has been documented in many insects, such as flies (Dweck et al., 2013). Typically, egg laying is accomplished through a sequential behavioral program. First, the pregnant females find a suitable substrate for laying eggs. Second, they use a specialized egg-laying appendage to make contact with the substrate and expulse eggs from the oviducts. Finally, the female animals expulse eggs out of the oviducts. Many genetic factors affecting female egg laying remain unclear. A complete understanding of egg laying can lead to the discovery of new pesticide targets for controlling pest populations.

The aim of this study was to explore whether TRPL plays a biological role in fertility of the fruit fly and the brown planthopper. In this study, we have identified and characterized the *Nltrpl* gene, which expresses TRPL in oviducts of brown planthoppers. Knocking down *Nltrpl* using RNA interference led to a significant decrease in *Nlvg* and *Nlvgr* mRNA levels and egg production compared to controls. Remarkably, similar egg-laying defects were observed in *trpl* mutant flies. The fly mutants could be restored to normal by expressing *Nltrpl*, suggesting TRPL is functionally conserved between flies and brown planthoppers. In flies, TRPL is selectively expressed in a subset of neurons in the oviduct. Inactivation of these neurons caused impairments in egg laying, indicating that TRPL-expressing neurons are required for egg laying. In summary, we uncovered a role of TRPL in egg laying in both flies and brown planthoppers. In light of an indispensable function of TRPL in the regulation of brown planthopper reproduction and multiplication, our work indicates that the TRPL protein is an excellent target for new insecticide development for brown planthopper control.

## Results

### Sequence and Phylogenetic Analyses of *Nltrpl*

**FIGURE 1:**
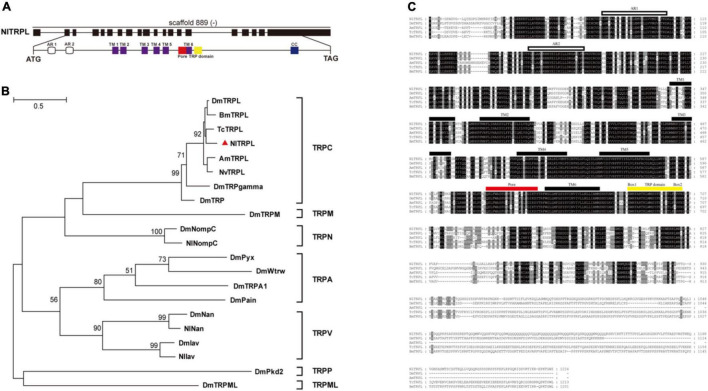
Exon-intron organization in *Nltrpl* gene and phylogenetic analysis of NlTRPL and TRP family. **(A)** Exons are shown as black boxes. The lines between boxes represent introns. The predicted start codon (ATG), stop codon (TAG), and scaffold of the gene locus (“–” represents the opposite orientation with the scaffold) are also shown in their corresponding positions. The detail below shows the functional domains of *Nltrpl*. White cylinders represent ankyrin repeats (AR); purple boxes, transmembrane domain (TM); red box, pore structure; yellow box, TRP domain (EWKFAR and LPPPFN); blue box, coiled coil (CC). **(B)** Phylogenetic tree created using the maximum likelihood method in MEGA 5.2.2 software. The numbers at the nodes of the branches represent the numbers of bootstrap replications supporting that branch. The tree includes TRPL in *Nilaparvata lugens* (Nl, identified by the triangle), *Drosophila melanogaster* (Dm), *Bombyx mori* (Bm), *Tribolium castaneum* (Tc), *Apis mellifera* (Am), and *Nasonia vitripennis* (Nv), as well as other TRP channels in *Drosophila*. **(C)** Amino acid sequence alignment of NlTRPL and TRPL orthologs from *Drosophila melanogaster* (Dm), *Apis mellifera* (Am), *Tribolium castaneum* (Tc), and *Bombyx mori* (Bm). The amino acid position is shown on the right. Identical residues among orthologous sequences are shown as white characters against a black background, and conservative substitutions are shown as gray shading. Black rectangles above the sequence represent the transmembrane domain (TM); white rectangles representing the ankyrin repeat (AR); the red rectangle representing the pore domain; the yellow rectangle representing the TRP domain. The accession numbers of the sequences used are listed in Supplementary Table 2.

Based on the amino acid sequence of the TRPL protein of *Drosophila*, the full-length sequence of *Nltrpl* was cloned. The *Nltrpl* gene, located on scaffold 889 of the brown planthopper genome, consists of 19 exons spanning approximately 58 kb of genomic DNA. Its full-length cDNA contains a 3,672 bp open reading frame encoding 1,224 amino acids (Figure 1A) (GenBank accession no. KX249689). Similar to the TRPC subfamily in other species, NlTRPL has a TRP domain at the C-terminus (Figure 1A). Phylogenetic tree analyses show that NlTRPL clustered with TcTRPL, BmTRPL, DmTRPL, and AmTRPL (Figure 1B). Amino acid sequence comparisons between the brown planthopper and other insects showed that NlTRPL has 85, 78, 78, and 76% identity with the TRPL proteins of *Tribolium castaneum*, *Bombyx mori*, *Drosophila melanogaster*, and *Apis mellifera* (Figure 1C).

### Expression Patterns of *Nltrpl*

**FIGURE 2:**
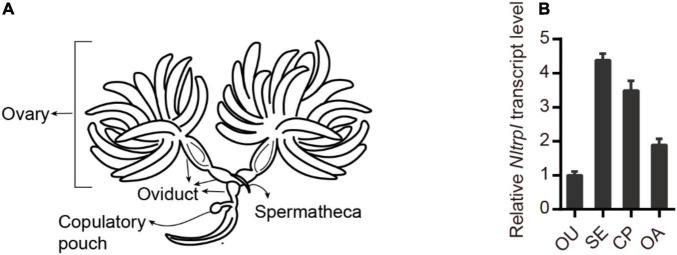
Expression patterns of *Nltrpl* in the female reproductive organ. **(A)** A diagram showing the reproductive organ of female brown planthoppers. **(B)** Expression patterns of *Nltrpl* in the four female reproductive organ regions: oviduct (OU), spermatheca (SE), copulatory pouch (CP), and ovary (OA). Data are expressed as mean ± SEM.

To characterize the spatial and temporal expression patterns of *Nltrpl* in the reproductive organ, we performed quantitative real-time PCR (qRT-PCR) analyses of the female reproductive organ (Figure 2A). *Nltrpl* exhibits prominent expression in different parts of the female reproductive system, including the spermatheca, copulatory pouch, and ovary (Figure 2B). In addition, *Nltrpl* is expressed in many developmental stages, including eggs, first- through fifth-instar nymphs, and adults (males and females with brachypterous and macropterous) (Supplementary Figure 1). *Nltrpl* shows higher expression in adults than in other developmental stages, implying that *Nltrpl* exhibits an essential physiological function in the adult stage.

### Knocking Down *Nltrpl* Leads to a Significant Reduction in Egg Laying

**FIGURE 3:**
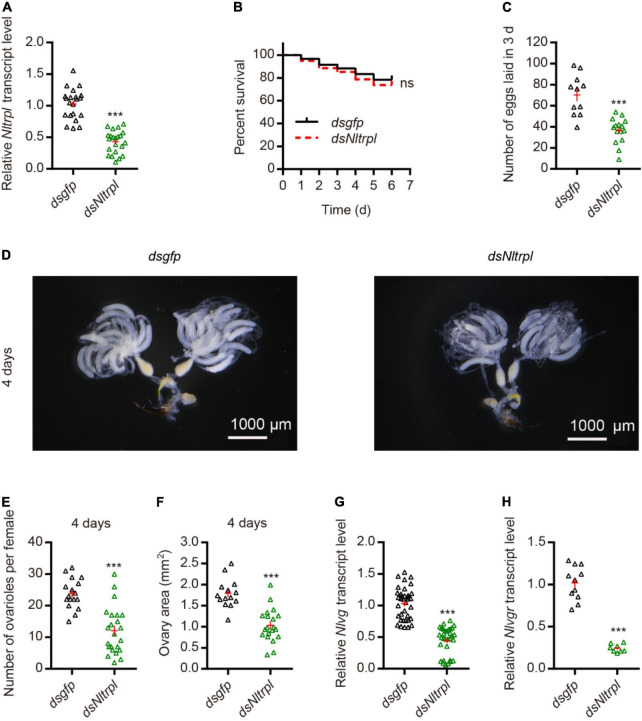
Knockdown of *Nltrpl* in the brown planthopper (BPH) impaired egg-laying behavior. **(A)** Downregulation of *Nltrpl* in BPH using *dsNltrpl* reduced mRNA expression levels compared to BPH injected with *dsgfp*. **(B)** Survival rate of BPH injected with *dsgfp* (*n* = 60) and *dsNltrpl* (*n* = 60). Survival analyses with log-rank test: χ^2^ = 0.3669, not significant (ns). **(C)** Number of eggs laid during the 3 days after *dsgfp* or *dsNltrpl* injection. Underdeveloped ovaries **(D)** and detained eggs per ovary **(E)** per female 4 days after injection of *dsgfp* or *dsNltrpl*. **(F)** Ovary area in per female 4 days after injected with *dsgfp* or *dsNltrpl*. mRNA expression level of BPH vitellogenin gene [*Nlvg*; **(G)**] and vitellogenin receptor gene [*Nlvgr*; **(H)**] after injection with *dsgfp* or *dsNltrpl*. ***P < 0.001, Student’s *t*-test.

To investigate the role played by *Nltrpl* in female fecundity, we synthesized the double-stranded RNA (dsRNA) against *Nltrpl* (*dsNltrpl*) fragment *in vitro* and injected it into brown planthoppers to knock down *Nltrpl* expression levels. Injection of *dsNltrpl* in females successfully knocked down the target gene, with 60% suppression (Figure 3A), indicating that this specific *dsNltrpl* fragment effectively inhibited the transcription level of *Nltrpl*. To rule out the effect of mortality on egg production, we calculated the 6-day survival rate of brown planthoppers after dsRNA injection; the results showed that female survival was not affected by *Nltrpl* dsRNA (Figure 3B). Notably, *dsNltrpl*-treated females produced considerably fewer eggs than control females treated with GFP (*dsgfp*) (Figure 3C). Compared to *dsgfp*-treated females, brown planthoppers injected with *dsNltrpl* had underdeveloped ovaries (Figure 3D), fewer detained eggs per ovary, and smaller ovary size (Figures 3E,F). Vitellogenin (Vg) and vitellogenin receptor (Vgr) are typically used as molecular markers to monitor fecundity in different insects, including brown planthoppers (Qiu et al., 2016; Pang et al., 2017). Thus, we measured mRNA expression levels of *Nlvg* and *Nlvgr* in the females, which were decreased significantly after inhibition of *Nltrpl* expression (Figures 3G,H). These results indicate that *Nltrpl* is required for the expression or stability of *Nlvg* and *Nlvgr* mRNA transcripts.

### Expression Pattern of Transient Receptor Potential-Like in Flies

**FIGURE 4:**
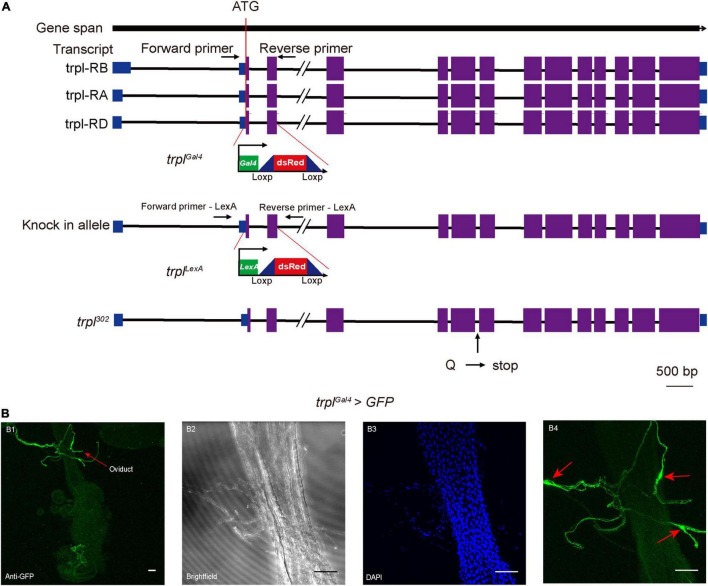
Expression patterns of TRPL on the reproductive organ of female *Drosophila*. **(A)** Generation of the *trpl* reporter and mutant alleles. **(B)** Expression of UAS-mCD8-GFP in sensory neurons innervating the female oviduct under control of *trpl^Gal4^* (*trpl* > *mCD8-GFP*) **(B1–B4)**. Arrows in **(B1,B4)** indicate the somas of sensory neurons. Scale bars, 50 μm.

Since genetic manipulation of the brown planthopper has not been established, we took advantage of genetic tools in *Drosophila* to study whether *trpl* participates in egg laying in insects. We utilized the CRISPR/Cas9 gene-editing technique *via* ends-out homologous recombination (Gong and Golic, 2003) to generate the *Gal4* and *LexA* knock-in mutants *trpl^Gal4^* and *trpl^LexA^* (Figure 4A). The *Gal4* or *LexA* gene with the *dsRed* gene was inserted into the *trpl* locus near the translation initiation codon of the *Drosophila trpl* so that about 437 bp of the *trpl* gene was replaced by the reporter construct (Supplementary Figure 3). Consequently, more than 145 amino acids, about one-fifth of the coding sequence of *trpl*, were deleted in the *trpl^Gal4^* and *trpl^LexA^* alleles, resulting in a loss of function of the *trpl* gene. This was confirmed by RT-PCR, which detected no *trpl* genomic DNA in *trpl^LexA^* and *trpl^Gal4^* mutants (Supplementary Figure 3).

To determine the expression pattern of *trpl*, we used *trpl^Gal4^* to drive the expression of the mCD8-GFP reporter. Our results revealed that *trpl* is expressed in sensory neurons innervating the female oviduct (Figures 4B1–B4). Surprisingly, *trpl* was not expressed in the fly ovary (Supplementary Figure 4B). Consistent with previous work (Zhang et al., 2013), *trpl* was also detected in a subset of regions in the brain, including the optic lobe (Supplementary Figure 4A). The reporter also stained the subesophageal ganglion (Supplementary Figure 4A) and gustatory receptor neurons at the labellum (Supplementary Figure 4C).

### Transient Receptor Potential-Like Is Required for Egg Laying in Flies

**FIGURE 5:**
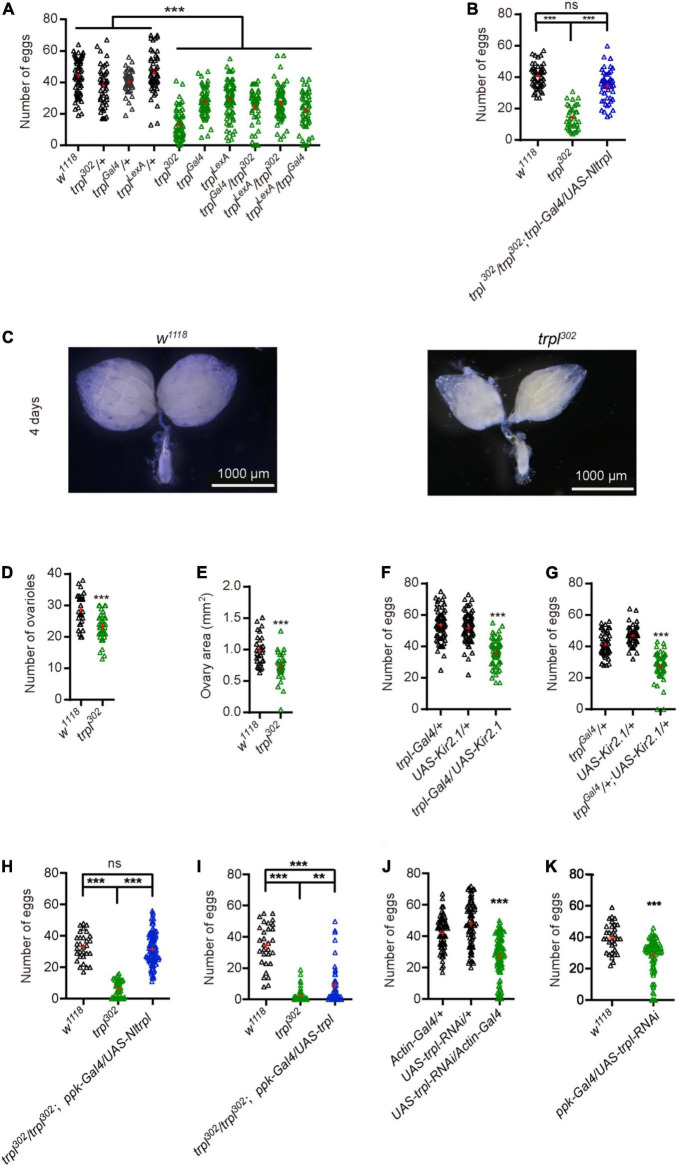
Transient receptor potential-like (*trpl*) is essential for fruit fly egg-laying behavior. **(A)** Numbers of eggs laid by wild-type and *trpl* mutant flies. **(B)** The *trpl* mutants were rescued by expressing *Nltrpl*. **(C)** Ovary development of wild-type (*w*^1118^) and *trpl*^302^ mutants 4 days after eclosion. Ovariole number **(D)** and ovary area **(E)** in wild-type and mutant files. Number of eggs was reduced after *trpl*-expressing neurons were inhibited using *Kir2.1 via trpl*-*Gal4* **(F)** or *trpl^Gal4^* **(G)**. **(H,I)** Rescue of *trpl* mutants with a *ppk-Gal4* that expressed in oviduct neurons. **(J)** Number of eggs from controls and mutant flies in which *trpl* is globally knocked down. **(K)** Number of eggs from wild type and mutant flies with *trpl* selectively knocked down in oviduct sensory neurons. ***P < 0.001, one-way ANOVA with Tukey’s multiple comparisons test.

We sought to determine whether the *trpl* gene is involved in fly egg laying. Egg-laying assays showed that the number of eggs of *trpl* mutants was significantly reduced compared with control flies (Figure 5A). To test whether *Nltrpl* can rescue the egg-laying defects of fly *trpl* mutants, we reintroduced a wild-type *Nltrpl* gene into the *trpl*^302^ mutants using *trpl*-*Gal4*. We found that expression of *Nltrpl* can restore the *trpl*^302^ mutant to egg laying equivalent to that of wild-type animals (Figure 5B). Therefore, our data demonstrate that NlTRPL can functionally replace fly TRPL, indicating an evolutionarily conserved role of TRPL in egging laying.

Furthermore, we examined ovary development in fruit flies and found that in *trpl* mutants ovaries developed abnormally (Figure 5C). Ovariole number and ovary size in *trpl* mutants were smaller compared to controls (Figures 5D,E). Surprisingly, we detected no TRPL expression in the fly ovary (Supplementary Figure 4B). Thus, we speculate that TRPL indirectly participates in ovary development by a cell non-autonomous mechanism.

Females use a variety of sensory cues to determine the feasibility of egg deposition (Karageorgi et al., 2017). We hypothesize that *trpl*-expressing neurons in the oviduct may sense the stimulation of the external or internal environment and relay signals to the central nervous system to trigger egg laying. To determine whether the *trpl*-expressing neurons in the oviduct are required for egg laying, we selectively inactivated the output of *trpl*-expressing neurons by using *trpl*-*Gal4* or *trpl^Gal4^* to direct expression of Kir2.1 (Sweeney et al., 1995). Inactivation of *trpl*-expressing neurons caused fruit flies to lay fewer eggs than controls (Figures 5F,G), suggesting that the neuronal activity of *trpl*-expressing neurons are required for egg laying.

Based on our immunocytochemical assays, the *trpl*-expressing neurons associated with the oviduct are sensory neurons. To test whether *Nltrpl* can rescue the egg-laying defects of fly *trpl* mutants, we selectively express *trpl* in fly oviduct sensory neurons using the *ppk-Gal4*, which labels sensory neurons in the oviduct (Grueber et al., 2007; Ameku and Niwa, 2016). We found that expression of *Nltrpl* in fly oviduct sensory neurons can fully restore the egg-laying impairments observed in *trpl*^302^ mutant to a normal level (Figure 5H). Furthermore, expressing a wild-type fly *trpl* gene back into *trpl*^302^ mutants led to a partial rescue (Figure 5I). Therefore, our results indicate that TRPL mainly functions in the oviduct sensory neurons to regulate egg laying. We also knocked down the neurons that overlap with those same TRPL-expressing neurons. When knocking down *trpl* neurons by *actin-Gal4*. Egg-laying assays showed that the number of eggs produced by the mutant flies with *trpl* globally knocked down by the *actin-Gal4* was significantly reduced compared with control flies (Figure 5J). Of great interest, our data demonstrate that knocking down *trpl* using *ppk-Gal4* also leads to egg-laying impairments as compared to controls (Figure 5K).

In conclusion, our results demonstrate that *trpl* is essential for egg laying in both the brown planthopper and the fruit fly. Our findings identify a novel function of TRPL, which can be used as potential target to develop new insecticides.

## Discussion

In this study, we identified the *trpl* gene of the TRPC subfamily in the brown planthopper (Figure 1). Based on full-length cDNA, *Nltrpl* is highly similar with other TRPL proteins reported in insects. Members of the TRPC subfamily contain six transmembrane segments (TM1 to TM6) with a putative pore region (Yue et al., 2015). Like other TRPC subfamily members, the TRPL channel of brown planthoppers also contains multiple N-terminal ankyrin repeats and a TRP domain after the sixth transmembrane segment (Vennekens et al., 2002). The fly TRPL has four Ars. In contrast, the SMART module analysis of brown planthopper TRPL revealed that NlTRPL only has two Ars. Comparison of the amino acid sequences between brown planthoppers and the fruit fly shows that NlTRPL has 78% identify with the DmTRPL, supporting our finding that *Nltrpl* and *Dmtrpl* are functionally conserved in regulating egg-laying behavior (Figures 3, 5).

Egg laying is an important physiological process that is controlled by many genetic factors. Genes associated with this physiological process are usually selectively expressed in the female reproductive system. In the brown planthopper, a mucin-like protein (*NlESMuc*) that is specifically expressed in the egg chambers of the ovarioles reduces fecundity and causes lower egg production and difficult oviposition (Lou et al., 2019). The histone deacetylase (*NlHDAC*) shows high expression in the ovary and plays critical roles in female fertility by regulating development of the ovaries and ovipositor (Zhang et al., 2018). A β-adrenergic-like octopamine receptor (*OA2B2*) expressed in female reproductive regions is involved in egg-laying behavior (Wu et al., 2017). These genes are expressed in the female reproductive system with various expression patterns, which can regulate the egg laying (Li et al., 2015; Cury et al., 2019). In this study, we discovered that *trpl* is required for ovary development and fecundity, promoting ovulation of eggs. TRPL was expressed in female reproductive organs of flies. However, we detected no TRPL expression in the ovaries, suggesting that the TRPL-mediated signaling at the oviduct or other reproductive organs may influence ovary development. We speculate that the *trpl*-expressing neurons in the reproductive system may produce a paracrine signal to stimulate ovary development, an essential step prior to expelling the mature eggs through the oviduct. Consistent with this hypothesis, when we silenced *trpl*-expression neurons in fruit flies, their egg production decreased significantly. Their ovaries were not fully developed and the number of eggs deposited was very small (Figures 3, 5). We also test whether *trpl* can rescue the egg-laying defects of fly *trpl* mutants. When we express *trpl* in fly oviduct sensory neurons using the *ppk-Gal4*. We found that expression of *Nltrpl* can fully restore the egg-laying impairments (Figure 5H). However, expressing a wild type fly *trpl* back into *trpl*^302^ mutants led to a partial rescue (Figure 5I). There are a couple of reasons that can explain the partial rescue using the *ppk-Gal4* as a driver. First, the fly TRPL expression driven by the *ppk-Gal4* may be expressed at suboptimal levels as compared to endogenous TRPL levels. Second, there are three fly TRPL isoforms: TRPL-RA, RB, and RD. Since, we used only one isoform fly TRPL to do rescue, there are other isoforms of TRPL which may also be required in egg laying. All in all, our work strongly indicates that *trpl* is required for egg laying in both brown planthoppers and flies.

The brown planthopper is a highly disastrous pest, severely affecting rice harvests worldwide. The brown planthopper sucks the phloem sap and impales the rice epidermis with its ovipositor, thereby interfering with the normal function of transport tissue (Bao and Zhang, 2019). Unfortunately, the brown planthopper has developed resistance to almost all insecticides, including imidacloprid, thiamethoxam, and buprofezin over the past decade (Wu et al., 2018). Pymetrozine, an insecticide that targets the TRPV channel (Nesterov et al., 2015), has been the recommended insecticide for brown planthopper control (Zhang et al., 2014). Pymetrozine not only interferes with the feeding behavior of insects but also inhibits the fecundity of brown planthoppers (Wang et al., 2019a,b). Given this, our work suggests that TRPL could be an effective insecticide target to reduce brown planthopper crop damage by inhibiting egg laying and ovarian development.

## Materials and Methods

### Insects

The brown planthopper (BPH), *Nilaparvata lugens*, strain was provided by Zhejiang Research Institute of Chemical Industry (Hangzhou, Zhejiang, China) in 2005 and reared with rice seedlings at 27°C ± 1°C, relative humidity 70–80%, and 16-h light/8-h dark photoperiod.

Flies were raised at 25°C on standard medium. The *w*^1118^ fly strain was used as control. *Trpl*-*Gal4/TM6B* (BDSC: 24903), *UAS-Kir2.1* (FBtp0125506), and *trpl*^302^ mutants (BDSC: 24902) were obtained from the Bloomington *Drosophila* Stock Center (Bloomington, IN, United States). *UAS-trpl-RNAi* (TH02033.N) was purchased from Tsinghua Fly Center (Beijing, China).

### Cloning and Sequencing

TRIzol Reagent (Invitrogen, Carlsbad, CA, United States) was used to isolate total RNA from adult BPH ground with a grinder. The cDNA template was synthesized using the M-MLV reverse transcription kit (BioTeke, Beijing, China), and the synthesized cDNA template was stored at −20°C.

The amino acid sequences of TRPL proteins from *D. melanogaster* were obtained from Flybase.^1^ TBLASTN searched the *N. lugens* transcriptomic databases and genomic sequences (GenBank accession No. AOSB00000000) (Xue et al., 2014). The full-length gene was amplified by PCR using the forward primer Nltrpl-F (CTGGCTGGGCTGAGTGTTTTA) located upstream of the putative start codon initiator and the reverse primer Nltrpl-R (CCACAGGCCTGGATTTCAGT) located downstream of the putative stop codon. Following PCR amplification, the *trpl* fragment was purified and sequenced. The primer sequences used in this study are listed in Supplementary Table 1.

### Sequence and Phylogenetic Analysis

The exon and intron architectures of *Nltrpl* were predicted based on the alignments of putative open reading frames against their corresponding genomic sequences in Spidey^2^ and then structured on the website of GSDS v2.0^3^ (Hu et al., 2015). The transmembrane segments and topology of *Nltrpl* were predicted by TMHMM v2.0,^4^ and its ankyrin repeat domain organization was assessed using Simple Modular Architectural Research Tool (SMART).^5^

We used the BLAST service of the National Center for Biotechnology Information^6^ to conduct sequence alignment and analysis. Multiple alignments of *Nltrpl* were performed with the ClustalW Multiple Alignment program in BioEdit. The deduced amino acid sequence of *Nltrpl* was aligned with its corresponding orthologs from *Drosophila melanogaster* (NP_476895.1), *Bombyx mori* (XP_004922702.1), *Tribolium castaneum* (XP_968598.1), *Apis mellifera* (XP_006562675.1), and *Nasonia vitripennis* (XP_008203556.1) (Supplementary Table 2). We used the maximum likelihood method and bootstrapped with 1,000 replications in MEGA 5.2.2 software to construct the phylogenetic tree (Tamura et al., 2011).

### Quantitative Real-Time PCR

Total RNA was isolated from BPH using the TRIzol Reagent (Invitrogen, Carlsbad, CA, United States), including various developmental stages and tissue samples. Developmental stages sampled were eggs (*n* = 40–50); first-instar (*n* = 40–50), second-instar (*n* = 40–50), third-instar (*n* = 15–20), fourth-instar (*n* = 15–20), and fifth-instar nymphs (*n* = 10–15); and adults of both sexes and wing forms: brachypterous male (*n* = 10–15), brachypterous female (*n* = 10–15), macropterous male (*n* = 10–15), and macropterous female (*n* = 10–15). Fertilized eggs were collected 4 days after spawning. From nymphs to adults, the samples were collected every 24 h. Different development tissues were dissected from adult females, including ovary, oviduct, copulatory pouch, and spermatheca. First-strand cDNA was synthesized using an oligo (dT_18_) primer and 500 ng total RNA template in a 10 μl reaction according to the instructions of the HiScript^®^ II Q RT Super Mix synthetic qPCR (+gDNA wiper) kit (Vazyme, Nanjing, China).

The specific quantitative real-time PCR (qRT-PCR) primer sequences are listed in Supplementary Table 1. PCR was performed on a CFX96 real-time PCR detection system (Bio-Rad, Hercules, CA, United States). The reaction volume was 20 μl, including 4 μl 10-fold diluted cDNA, 1 μl each primer (10 μM), 10 μl 2 × Ultra SYBR mixture, and 4 μl RNase-free water. The qRT-PCR protocol was performed with the following cycling conditions: initial incubation of 95°C for 10 min; 40 cycles of 95°C for 15 s, 60°C for 40 s; followed by melting curve analysis. The expression level of *trpl* was normalized to 18S ribosomal RNA (*Nl18S*) (Xu et al., 2015). Quantitative variation was assessed using a relative quantification method (comparing threshold cycle, ^ΔΔ^Ct) (Livak and Schmittgen, 2001).

### Double-Stranded RNA Preparation and Injection

Following the manufacturer’s recommendation for the mMESSAGE mMACHINE T7 Transcription Kit (Ambion, Austin, TX, United States), the sequence of the fragment targeting 501 bp of the conserved region in the *trpl* genes (amino acids 615-782) was amplified by PCR with the forward primer 5′-TAATACGACTCACTATAGGGCTGTTTTGGGCCAGTTTC GG-3′ and reverse primer 5′-TAATACGACTCACTATAGGG GACGCATCGACGTGATCTCT-3′ and used as the template to synthesize *trpl* dsRNA (*dstrpl*). The synthesized dsRNA was evaluated for quantity and concentration by agarose gel electrophoresis and absorption spectroscopy. The product was dissolved in RNase-free water and stored in a −80°C freezer until use.

For microinjection, the Sutter Instruments P-97 micropipette puller (Sutter Instruments, Novato, CA, United States) was used to make the microscopic needle at the following settings: heat = 512, pull = 150, velocity = 30, time = 90. Microinjection of dsRNA was then performed using a previously described method (Zhang et al., 2015). The dsRNA product was added to the microinjection needle using a microloader. The fully mated female BPH were anesthetized with carbon dioxide for about 30 s, and 300 ng (about 60 nl) of dsRNA was injected into the membrane between the thorax and abdomen using an UltraMicro Pump II (UMP2) microinjector (World Precision Instruments, Sarasota, FL, United States). Approximately 150 adults were injected with *Nltrpl* dsRNA, and about 150 were injected with green fluorescent protein (GFP; *dsgfp*) to serve as controls. The experiment was repeated three times independently. We randomly selected brachypterous female adults to detect interference efficiency by qRT-PCR 3 days after injection.

### Egg Production and Survival Assay

To analyze female BPH egg production, we transferred five dsRNA-injected BPH females and five untreated males to a vial with fresh rice seedlings. At least 10 vials were observed for each treatment. After 3 days the number of eggs laid was counted under a stereomicroscope (Zeiss), and the females were recorded for survival.

Flies were kept in foil-covered food vials containing wet yeast paste. Then we placed 4- to 6-day-old individual flies in custom-made transparent Plexiglas egg-laying chambers (Gou et al., 2016), which were then placed in the dark. After 12 h, the total number of eggs was counted.

### Quantification of Ovary Size

Ovaries of mated female BPH and fruit flies were anatomized under a stereomicroscope (Zeiss). The ovaries were placed on a glass slide, and 50 μl PBS was added to float the ovaries to maintain their original shape. The ovary images were obtained by a light microscope with a digital video camera (Zeiss, ProgRes 3008 mF, Jenoptik, Jena, Germany) and were used to measure the area of individual ovaries and the number of eggs per ovary using the matching NIS-Elements software.

### Construction of *Drosophila* Mutants

For *UAS-Nltrpl* transgenic *Drosophila*, the *Nltrpl* cDNA was cloned into the pJFRC2-10XUAS-IVS-mCD8:*GFP* vector to generate the *UAS-Nltrpl* transgene. Subsequently, the eggs from PhiC31 source virgins crossed with (y1w67c23; P(CaryP) attP40) males were used for injection. Flies potentially containing modified alleles were selected against the marker phenotype (CyO) for the relevant balancer. All lines used were sequence verified.

For two *trpl* mutant flies (*trpl^Gal4^* and *trpl^LexA^*), the *Gal4* and *LexA* genes linked with *DsRed* were inserted into the *trpl* locus near the translation initiation codon of the *Drosophila trpl* and replaced a part of the coding sequence of the target gene by homologous recombination. The potential CRISPR targets in regions of *trpl* genomic sequences were identified in the online tool DRSC Find CRISPR.^7^ Two targets were selected to ligate into plasmids pCFD4-U6:1_U6:3tandemgRNAs (Addgene: 49411) to generate guide-RNA-expressing plasmids (Supplementary Figure 2 and Supplementary Table 1). We constructed donors’ plasmid pHD-DsRed-attp (Addgene: 51434) for homology-directed repair, and the 5′ and 3′ homologous arms of *trpl* were amplified from *w*^1118^ flies by PCR cloning. Each contained two ∼900 bp homology arms flanking the relevant CRISPR target region (Supplementary Figure 2). Primer sequences used in this study are listed in Supplementary Table 1. All injections were done by UniHuaii Co., Ltd. (Zhuhai, China).

### Tissue Dissection, Staining, and Imaging

Gut, labellum, and brain of 4- to 6-day-old female flies were dissected in Schneider’s insect medium and fixed in 4% paraformaldehyde in PBS for 25 min at room temperature. We used the standard antibody staining protocol to process the fixed tissues before mounting them with SlowFade Diamond (Life Technologies, Carlsbad, CA, United States). Samples were observed and photographed at 20 × or 60 × magnification on Zeiss 700 confocal microscopes (Zeiss, Jena, Germany) and a confocal laser scanning microscope (CLSM, LSM700, Zeiss) and then processed with ImageJ.

### Statistics

Each experiment was performed at least three different times. Experimental data were analyzed using GraphPad Prism 6 software (GraphPad Software Inc., San Diego, CA, United States). Student’s *t*-test, log-rank test, and Tukey’s multiple comparisons test were used to test differences between sets of normally distributed data.

## Data Availability Statement

The original contributions presented in this study are included in the article/Supplementary Material, further inquiries can be directed to the corresponding authors.

## Author Contributions

C-FG, YVZ, and S-FW: conceptualization. YZ: data curation and writing – original draft. C-FG: funding acquisition and supervision. YZ, Y-JZ, DG, L-XW, and C-DN: investigation. C-FG and YVZ: validation. C-FG, YVZ, L-XW, and S-FW: writing – review and editing. All authors contributed to the article and approved the submitted version.

## Conflict of Interest

The authors declare that the research was conducted in the absence of any commercial or financial relationships that could be construed as a potential conflict of interest.

## Publisher’s Note

All claims expressed in this article are solely those of the authors and do not necessarily represent those of their affiliated organizations, or those of the publisher, the editors and the reviewers. Any product that may be evaluated in this article, or claim that may be made by its manufacturer, is not guaranteed or endorsed by the publisher.

## Footnotes

1
http://flybase.org/

2
http://www.ncbi.nlm.nih.gov/spidey/

3
http://gsds.gao-lab.org/

4
http://www.cbs.dtu.dk/services/TMHMM-2.0/

5
http://smart.embl-heidelberg.de/

6
https://www.ncbi.nlm.nih.gov/

7
https://www.flyrnai.org/crispr/
