# Supplementary material for: Function of Transient Receptor Potential-Like Channel in Insect Egg Laying
Source: Front Mol Neurosci. 2022 Jun 30;15:823563. doi: 10.3389/fnmol.2022.823563 (PMC9280367; doi:10.3389/fnmol.2022.823563)
Supplement: Supplementary file 1 [file Data_Sheet_1.pdf]

Supporting information captions:

Table S1. The primers used in this study.

Primer name	Sequence (5′–3′)
For cDNA fragment cloning	
Nltrpl-F	CTGGCTGGGCTGAGTGTTTTA
Nltrpl-R	CCACAGGCCTGGATTTCAGT
For qRT-PCR	
QNltrpl-F	ACCCGAACATCCAGCAACTA
QNltrpl-R	TTTGCCGGTGTGTGTATTGG
QNl18S-F	CGCTACTACCGATTGAA
QNl18S-R	GGAAACCTTGTTACGACTT
QNltrpl-vg-F	GCTTGTCAGAATGCCACC
QNltrpl-vg-R	TCTTGCCAGAAGGATTGC
QNltrpl-vgr-F	ATCTACTTCACCGATTCTGG
QNltrpl-vgr-R	ATCACCGACCTGTTACCC
For dsRNA synthesis	
T7-Nltrpl-F	TAATACGACTCACTATAGGGCTGTTTTGGGCCAGTTTCGG
T7-Nltrpl-R	TAATACGACTCACTATAGGGGACGCATCGACGTGATCTCT
T7-gfp-F	TAATACGACTCACTATAGGGAAGGGCGAGGAGCTGTTCACCG
T7-gfp-R	TAATACGACTCACTATAGGGCAGCAGGACCATGTGATCGCGC
For transgenic flies	
Nltrpl-XhoI-UAS	TTCAGGCGGCCGCGGCTCGAGCAAAATGTCTGACGAGAAAAAGGA
Nltrpl-XbaI-UAS	CCTTCACAAAGATCCTCTAGACTAAATCCATCCGGTTTTTG
For mutant flies	
Dmtrpl-gRNA-F	TATATAGGAAAGATATCCGGGTGAACTTCGTCGCAAAAAGAAGCTG CCGAGTTTTAGAGCTAGAAATAGCAAG
Dmtrpl-gRNA-R	ATTTTAACTTGCTATTTCTAGCTCTAAAACGCTGGCTGTCGAGCGCG GCGCGACGTTAAATTGAAAATAGGTC
Dmtrpl-HR1-F	TCGCTGAAGCAGGTGGAATTCTCCCATTTCGATGGGCAGTC
Dmtrpl-HR1-EcoR1-LexA-R	TCTTCTTGGGTGGCATGAATTCTCTAGAGTTAGTGCACTTCACT
Dmtrpl-HR1-EcoR1-Gal4-R	ACTCAGCAGCTTCATGAATTCTCTAGAGTTAGTGCACTTCACT
Dmtrpl-HR2-Spe1-F	AAGTTATAGAAGAGCACTAGTTGGCTGTCGAGCGCGGCGATATGCC
Dmtrpl-HR2-Xho1-R	GATTGACGGAAGAGCCTCGAGCATCGTGCATGCCTCCAAAG
For confirm mutant flies	
$trpl^{LexA}$-F	AGTGAAGTGCACTAACTCTAGA
$trpl^{LexA}$-R	GGCATATCGCCGCGCTCGACAGCCA
$trpl^{Gal4}$-F	TGTGGCCCGGCTTAGTATTG
$trpl^{Gal4}$-R	ACCGCATCCAAAGAACCCAA

Table S2. Accession numbers of TRP amino acid sequences used in this study

Protein name	Accession number	Protein name	Accession number
TcTRPL	XP_9685981	DmTRPML	NP_6491451
NvTRPL	XP_0082035561	DmTRPA1	NP_6482635
BmTRPL	XP_0049227021	DmPain	NP_6119791
AmTRPL	XP_0065626751	DmPyx	NP_6120151
DmTRPL	NP_4768951	DmWtrw	NP_7311931
NlNompC	KX249691	DmNan	NP_6486962
NlNan	KX249697	DmIav	NP_5723531
NlIav	KX249698	DmNompC	ADK739851
DmTRPgamma	CAB962041	DmTRPM	NP_0010365481
DmPkd2	AAR240771	DmTRP	AAA289761

Figure S1

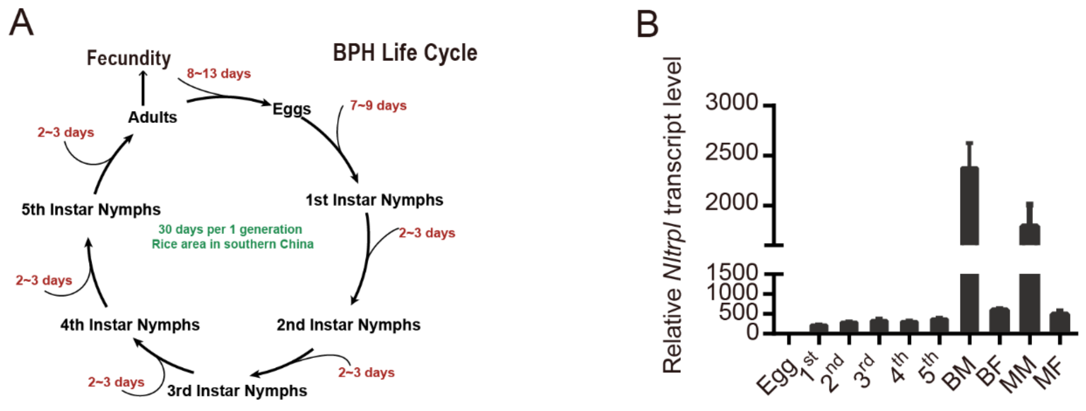

Figure S1. Expression patterns of *Nltrpl* in the brown planthopper (BPH) at different developmental stages. (A) Life cycle diagram of BPH. (B) Expression patterns of *Nltrpl* at different developmental stages: egg, first- to fifth-instar nymphs, and adult brachypterous males (BM) and females (BF) and macropterous males (MM) and females (MF).

Figure S2

```
TGATACTTCGATCGGTGCCAGGCAGAATTAACACATCTCGGCTGCAGAGGGAGAAATTAG 60
AGTGAAATATCATATGCATTTTACATCGTATGTTAACCGAATAGTTTATACTTGTTATAA 120
                      Donor HR1 →
TATATACAGGTGCAGATATAATCCCATTTCGATGGGCAGTCGCAGTATGCACCTGCTGTG 180
CAGGTGAACAGCTATAAATCCATTTATAAACGCTTCCCATGAAGTGGCGGATAAAACCGA 240
ATTTATTGCGACATTTTTATGTCCGTCCGCATTTTCGCCCGTCCTCCGCACTTATATTTC 300
TATTAAGTTAATTAAACAATGCACCATCGGGGCTCCTGGGGTAGTCAGAACCACCTTTTG 360
TTGGTACCGGAAGTGTTTTTTATGGAACAACCGCCATGATGATGGTTCCGAGTTTTTGCC 420
TGCCGCGCATACAAAGCACTTGCATTTTTTATGACCAGCCCAAACACTTTTAAATGCATC 480
TGCAGCATCCGCATCCATATCCGCATGCAGATCCGCATCCGCAGGAGCATAAAGCCAGTT 540
TAAAGCAAAAGCATGTAGACGGAATGCATTTTTTCTTTGCGGATTATCGAAAAAAGCTCC 600
TTAAATATTCATTTTGGCATTGTGTTCACACATGCGGAGGAAGGCAGCGGAAAATAAAAA 660
TATTTTCATCGGGTGTGGGAAAATATTGGGAAACAAAGAAATTAATTCTAATGGGAGAAA 720
CGATTGGCGATGATGTGAACAATCCAATGAGAGCAGCAACAACTAATACAAACTGCAGTT 780
CGTTGCTAATGAGATGAGTGCAATAAAAATGTAAATCAAAACAGAAAGCCGATTGAACAA 840
TTGGTCATAAGTGGTGCAATAAGTGCTCGCGAATTTGGTGTTCAGTGTGGCCTTTGGAAT 900
TGACCAGTAACAGTCGGGAAACAGTGAAACTGCACAGTGTCTAATCAACTCACTAAATTG 960
GCAAAACTTAAATCTTGCCCATGCAGATGGCACTTAAAAAGGGTTTAGCCAGCTCGGTCA 1020
GTGGCGGAAATAAAAAGAAAAACATTATAAACAGCTTAAATAGAAACTTTTTGCACGCCG 1080
GAAACCAGAAACCATAAACCAAGTCAAAAACCACCAGACACACACACACACACACCAACA 1140
CACACAAGCACACCCAGTTGGACATATAACTATGGACTATACAACTATGAGTACAAAAGC 1200
AAAATCAACTTGAAAGTTGCATGTGGCCCGGCTTAGTATTGGACTTTTTTGGCTTTGCCA 1260
TTGTGTATATAAATATATATGCCATATATGTAAATATCGTTCACTTTTCACTGCAAGTGT 1320
GCAAAGCCCTGCGACATGAAGTCGGATTTTGAACTCTGAACCCTGGACTTGGTACATGGA 1380
                                               ← Donor HR1    gRNA1
ATACTAGTTACGAACTGCTGGCGTTCGGTTAAGTGAAGTGCACTAACTCTAGAATGGGTC 1440
                     PAM
GCAAAAAGAAGCTGCCGACGGGCGTAAGTAGAAACCCATCCAGGATCCCGATCCAGATCC 1500

GTATCCAGCAAGTAGCATGTCTTAAAGCCGCCAGTATTAGCTAAACGCCGCTTACCTTAA 1560
ACGCCTCCAATGCCATTTAGATGGAAGCCAGACAATCAGCCCAGCATCCTCCACTTTGCA 1620
CTTAATTTTCTTGTATGTTTATTTTTTATTTATTTGCAATTCCGCATACACGATTTCCCC 1680
CTCGCCACATCGGCATGTACGTATGTGGCTCCTGCTCACCTGTCTCTTTAAATTATGCCA 1740
GGTATCGTCTGGCGTCAGCCACGCCTCCTCCGCCCCCAAATCGGTGGGCGGGTGCTGTGT 1800
                                                 PAM gRNA2
GCCGCTGGGATTACCACAGCCTCTGCTGCTCGAGGAGAAGAAGTTCCTGCTGGCTGTCGA 1860
                                                      Donor HR2 →
GCGCGGCGATATGCCGAATGTGCGCAGGTGAGTAGCGCTTTTCACGCCTCCGATGCCCAG 1920
CAGGGAGGGGGGGGGGGTGGGTGGTGTTTCTGTATGGCATCTTGGGCGGGAGGGATACAC 1980
TGCAAGAAACTCTTACCACAATCAGGTGTTGGATGAGCATTAGTATTCAAAGTTAATGTA 2040
CAAGCTTAGCTAATGAATGTATCAATCGAGTAGAACCTAATGTATATTGATATATTTAGC 2100
CTTCAATTTAAAAGAAATAGTTAAACACACTACCACATTTATCCGAGTGTAGGGTGTTCG 2160
AATAAAAACCCTGACATTTTTAGAGGGGTATCACGAGCTCCAATGGTGGCTTCGCCCAGG 2220
GTAACACAGGTGTTGACTGGGCTGGGTCGCACAGGTACCATAAATCCCATCGGGAGCCTC 2280
CTTGGGTTCTTTGGATGCGGTGCTGGTGGTGGGTTCAATTATTTGCACACTCGTCTGCCG 2340
CGGGCCTCAAAATGGTTACCTAACTGGCATCCCGGCAGCCACTTAATAAGCAATATGAGG 2400
CCTTCGACCCGGATACTTTACGCTCGTTTACGGCTTTGCTTTAACAAATGTACGATTAAT 2460
TCCGTTTGCTGGCAATGAATAAGAGATAAGCCAAATTATCCAAATACTTTTCGGTTGCCA 2520
CTTAAAGCTGTTAACATGCACAATCAATTCGAGAAACGAATCTTATTGAAAAGCAACTTT 2580
TCGGATAACTTAAGTTCCCTTACCTGAAAGCACAACTAAACCTTACTTTTTGTTGACATC 2640
CCAGGATGTCATTTATGAACAATTCTTTATTGTTTTCATCGGGCGAGTGATACGGTAACA 2700
ACAAGCAAATGAGCCGTGTCCGGGGTTCCTGTTTCATTTGTGATTCCCAATACGCTTTGG 2760
AGGCATGCACGATGTCCATATAATCTTGCATCTAATGTGAAATTGTGGAAGTAGTGGCGA 2820
  ← Donor HR2
```

Figure S2. *Drosophila* genome modification strategy. The CRISPR/Cas9 targets are shown in

gray, with the corresponding PAM (–NGG) triplets marked. CRISPR/Cas9-induced double-stranded breaks are indicated by vertical red lines.

Figure S3

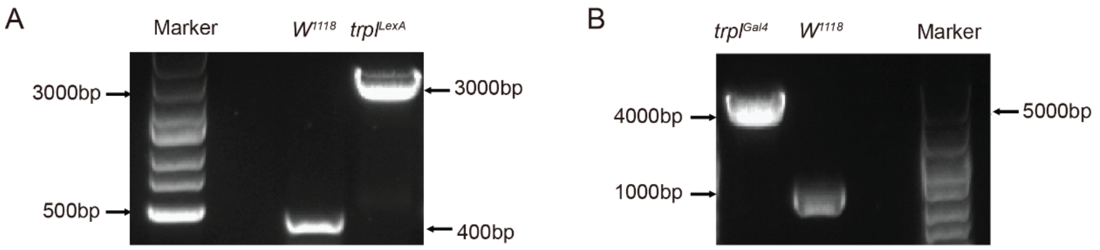

Figure S3. RT-PCR confirmed *Drosophila* mutants *trpl*^{LexA} (A) and *trpl*^{Gal4} (B).

Figure S4

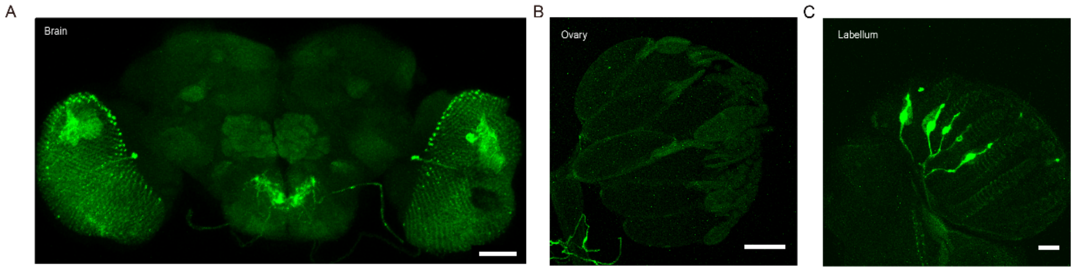

Figure S4. Expression of *Drosophila* UAS-mCD8-GFP in brain (A), ovary (B), labellum (C) under control of *trpl*^{Gal4} (*trpl>mCD8-GFP*). Scale bars: 200 µm (A and B), 20 µm (C).
